# Supplementary material for: Deriving Lipid Classification Based on Molecular Formulas
Source: Metabolites. 2020 Mar 24;10(3):122. doi: 10.3390/metabo10030122 (PMC7143220; doi:10.3390/metabo10030122)
Supplement: Supplementary file 1 [file metabolites-10-00122-s001.pdf]

Supporting Information for

Deriving Lipid Classification based on Molecular Formula

Joshua M. Mitchell [1,2,3,4], Robert M. Flight [1,2,3,5] and Hunter N.B. Moseley [1,2,3,6,7,*]

[1] Department of Molecular & Cellular Biochemistry, University of Kentucky, Lexington, KY USA
[2] Markey Cancer Center, University of Kentucky, Lexington, KY USA
[3] Resource Center for Stable Isotope Resolved Metabolomics, University of Kentucky, Lexington, KY USA
[4] jmmi243@uky.edu
[5] robert.flight@uky.edu
[6] Institute for Biomedical Informatics, University of Kentucky, Lexington, KY USA
[7] Center for Clinical and Translational Science, University of Kentucky, Lexington, KY USA
* Correspondence: hunter.moseley@uky.edu

Table of Contents

Content	Page
Cover Page	S-1
Table of Contents	S-2
Table S1: Isomeric Species per Lipid Category and Class (LMSD)	S-3
Table S2: Isomeric Species per Lipid Category and Class (LMSD+LMISSD)	S-6
Table S3: LMSD + HMDB_non_Lipid Model Performance (Classes)	S-10
Table S4: LMSD + LMISSD HMDB_non_Lipid Model Performance (Classes)	S-13
Table S5: LMSD + HMDB_non_lipid Model Performance on CH (Classes)	S-16
Table S6: LMSD + LMISSD + HMDB_non_lipid Model Performance on CH (Classes)	S-19
Figure S1: Training Set Mass Distributions	S-22
Figure S2: Cross-Sample Correspondence with LC at E-value <= 0.01	S-23
Figure S3: Cross-Sample Correspondence with LC at E-value <= 0.5	S-24
Figure S4: Cross-Sample Correspondence at E-value <= 0.01	S-25
Figure S5: Cross-Sample Correspondence at E-value <= 0.1	S-26
Figure S6: Cross-Sample Correspondence at E-value <= 0.5	S-27

Supplemental Table 1: Isomeric Species per Lipid Category and Class (LMSD)

LMSD Category or Class	Total Number of Entries	Total Number of Non-Isomeric Entries
Fatty Acyls [FA]	8830	1659
Glycerolipids [GL]	7542	533
Glycerophospholipids [GP]	9629	1698
Polyketides [PK]	6803	1345
Prenol Lipids [PR]	1251	469
Saccharolipids [SL]	1316	102
Sphingolipids [SP]	4357	1393
Sterol Lipids [ST]	2828	712
Other Fatty Acyls [FA00]	21	19
Fatty alcohols [FA05]	440	151
Fatty Acids and Conjugates [FA01]	2406	616
Octadecanoids [FA02]	567	39
Eicosanoids [FA03]	1115	136
Acyltrehaloses [SL03]	1297	83
Docosanoids [FA04]	1126	18
Fatty aldehydes [FA06]	254	89
Fatty esters [FA07]	1557	514
Fatty amides [FA08]	279	201
Fatty nitriles [FA09]	2	2
Fatty ethers [FA10]	3	2
Hydrocarbons [FA11]	698	121
Oxygenated hydrocarbons [FA12]	289	86
Fatty acyl glycosides [FA13]	71	53
Other Glycerolipids [GL00]	9	8
Monoradylglycerols [GL01]	27	19
Diradylglycerols [GL02]	570	163
Triradylglycerols [GL03]	6883	314
Glycosylmonoradylglycerols [GL04]	8	3
Glycosyldiradylglycerols [GL05]	45	30
Other Glycerophospholipids [GP00]	16	15
Glycerophosphocholines [GP01]	1768	320
Glycerophosphoethanolamines [GP02]	1316	260
Glycerophosphoinositols [GP06]	1191	236
Glycerophosphoserines [GP03]	1190	239
Glycerophosphoglycerols [GP04]	1212	250
Glycerophosphoglycerophosphates [GP05]	4	3
Glycerophosphoinositol monophosphates [GP07]	4	2
Glycerophosphoinositol bisphosphates [GP08]	3	1
Glycerophosphoinositol trisphosphates [GP09]	1	1

Glycerophosphates [GP10]	1203	242
Glyceropyrophosphates [GP11]	2	2
Glycerophosphoglycerophosphoglycerols [GP12]	1307	75
CDP-Glycerols [GP13]	38	19
Glycosylglycerophospholipids [GP14]	7	6
Glycerophosphoinositolglycans [GP15]	337	205
Glycerophosphonocholines [GP16]	1	1
Glycerophosphonoethanolamines [GP17]	1	1
Di-glycerol tetraether phospholipids (caldarchaeols) [GP18]	1	1
Glycerol-nonitol tetraether phospholipids [GP19]	1	1
Oxidized glycerophospholipids [GP20]	26	17
Other Polyketides [PK00]	1	1
Linear polyketides [PK01]	7	7
Halogenated acetogenins [PK02]	2	1
Annonaceae acetogenins [PK03]	3	1
Macrolides and lactone polyketides [PK04]	41	38
Ansamycins and related polyketides [PK05]	8	8
Polyenes [PK06]	5	4
Linear tetracyclines [PK07]	8	8
Angucyclines [PK08]	1	1
Polyether antibiotics [PK09]	35	10
Aflatoxins and related substances [PK10]	6	6
Cytochalasins [PK11]	3	3
Flavonoids [PK12]	6533	1169
Aromatic polyketides [PK13]	81	69
Non-ribosomal peptide/polyketide hybrids [PK14]	8	7
Phenolic lipids [PK15]	61	45
Isoprenoids [PR01]	1148	382
Quinones and hydroquinones [PR02]	38	36
Polyprenols [PR03]	34	30
Hopanoids [PR04]	31	28
Acylaminosugars [SL01]	9	9
Acylaminosugar glycans [SL02]	3	3
Other acyl sugars [SL05]	8	8
Other Sphingolipids [SP00]	19	17
Sphingoid bases [SP01]	88	67
Ceramides [SP02]	185	101
Phosphosphingolipids [SP03]	328	144
Phosphonosphingolipids [SP04]	3	3
Neutral glycosphingolipids [SP05]	2173	554
Acidic glycosphingolipids [SP06]	1558	521
Basic glycosphingolipids [SP07]	1	1
Amphoteric glycosphingolipids [SP08]	2	2
Sterols [ST01]	972	350

Steroids [ST02]	242	93
Secosteroids [ST03]	744	208
Bile acids and derivatives [ST04]	711	75
Steroid conjugates [ST05]	159	101

Supplemental Table 2: Isomeric Species per Lipid Category and Class (LMSD+LMISSD)

LMSD or LMISSD Category or Class	Total Number of Entries	Total Number of Non-Isomeric Entries
Fatty Acyls [FA]	10593	1659
Glycerolipids [GL]	795212	2506
Glycerophospholipids [GP]	313768	8039
Polyketides [PK]	6803	1345
Prenol Lipids [PR]	1251	469
Saccharolipids [SL]	1316	102
Sphingolipids [SP]	41891	3078
Sterol Lipids [ST]	2828	712
Other Fatty Acyls [FA00]	21	19
Fatty alcohols [FA05]	440	151
Fatty Acids and Conjugates [FA01]	2406	616
Octadecanoids [FA02]	759	39
Eicosanoids [FA03]	1639	136
Acyltrehaloses [SL03]	1297	83
Docosanoids [FA04]	2173	18
Fatty aldehydes [FA06]	254	89
Fatty esters [FA07]	1557	514
Fatty amides [FA08]	279	201
Fatty nitriles [FA09]	2	2
Fatty ethers [FA10]	3	2
Hydrocarbons [FA11]	698	121
Oxygenated hydrocarbons [FA12]	289	86
Fatty acyl glycosides [FA13]	71	53
Other Glycerolipids [GL00]	9	8
Monoradylglycerols [GL01]	193	62
Diradylglycerols [GL02]	16506	493
Triradylglycerols [GL03]	757784	1243
Glycosylmonoradylglycerols [GL04]	8	3
Glycosyldiradylglycerols [GL05]	20712	911
Other Glycerophospholipids [GP00]	16	15
Glycerophosphocholines [GP01]	11029	572
Glycerophosphoethanolamines [GP02]	10555	529
Glycerophosphoinositols [GP06]	10430	515

Glycerophosphoserines [GP03]	10429	518
Glycerophosphoglycerols [GP04]	10451	526
Glycerophosphoglycerophosphates [GP05]	9243	513
Glycerophosphoinositol monophosphates [GP07]	28053	617
Glycerophosphoinositol bisphosphates [GP08]	27471	509
Glycerophosphoinositol trisphosphates [GP09]	9157	509
Glycerophosphates [GP10]	10442	520
Glyceropyrophosphates [GP11]	2	2
Glycerophosphoglycerophosphoglycerols [GP12]	131628	316
CDP-Glycerols [GP13]	38	19
Glycosylglycerophospholipids [GP14]	7	6
Glycerophosphoinositolglycans [GP15]	337	205
Glycerophosphonocholines [GP16]	1	1
Glycerophosphonoethanolamines [GP17]	1	1
Di-glycerol tetraether phospholipids (caldarchaeols) [GP18]	1	1
Glycerol-nonitol tetraether phospholipids [GP19]	1	1
Oxidized glycerophospholipids [GP20]	44476	3882
Other Polyketides [PK00]	1	1
Linear polyketides [PK01]	7	7
Halogenated acetogenins [PK02]	2	1
Annonaceae acetogenins [PK03]	3	1
Macrolides and lactone polyketides [PK04]	41	38
Ansamycins and related polyketides [PK05]	8	8
Polyenes [PK06]	5	4
Linear tetracyclines [PK07]	8	8
Angucyclines [PK08]	1	1
Polyether antibiotics [PK09]	35	10
Aflatoxins and related substances [PK10]	6	6
Cytochalasins [PK11]	3	3
Flavonoids [PK12]	6533	1169
Aromatic polyketides [PK13]	81	69
Non-ribosomal peptide/polyketide hybrids [PK14]	8	7
Phenolic lipids [PK15]	61	45
Isoprenoids [PR01]	1148	382
Quinones and hydroquinones [PR02]	38	36
Polyprenols [PR03]	34	30
Hopanoids [PR04]	31	28
Acylaminosugars [SL01]	9	9

Acylaminosugar glycans [SL02]	3	3
Other acyl sugars [SL05]	8	8
Other Sphingolipids [SP00]	19	17
Sphingoid bases [SP01]	88	67
Ceramides [SP02]	9441	689
Phosphosphingolipids [SP03]	12808	676
Phosphonosphingolipids [SP04]	3	3
Neutral glycosphingolipids [SP05]	17971	1119
Acidic glycosphingolipids [SP06]	1558	521
Basic glycosphingolipids [SP07]	1	1
Amphoteric glycosphingolipids [SP08]	2	2
Sterols [ST01]	972	350
Steroids [ST02]	242	93
Secosteroids [ST03]	744	208
Bile acids and derivatives [ST04]	711	75
Steroid conjugates [ST05]	159	101
Fatty Acyls [FA]	10593	1659
Glycerolipids [GL]	795212	2506
Glycerophospholipids [GP]	313768	8039
Polyketides [PK]	6803	1345
Prenol Lipids [PR]	1251	469
Saccharolipids [SL]	1316	102
Sphingolipids [SP]	41891	3078
Sterol Lipids [ST]	2828	712
Other Fatty Acyls [FA00]	21	19
Fatty alcohols [FA05]	440	151
Fatty Acids and Conjugates [FA01]	2406	616
None	1	1
Octadecanoids [FA02]	759	39
Eicosanoids [FA03]	1639	136
Acyltrehaloses [SL03]	1297	83
Docosanoids [FA04]	2173	18
Fatty aldehydes [FA06]	254	89
Fatty esters [FA07]	1557	514
Fatty amides [FA08]	279	201
Fatty nitriles [FA09]	2	2
Fatty ethers [FA10]	3	2
Hydrocarbons [FA11]	698	121
Oxygenated hydrocarbons [FA12]	289	86

Fatty acyl glycosides [FA13]	71	53
Other Glycerolipids [GL00]	9	8
Monoradylglycerols [GL01]	193	62

Tables S1 and S2:

Lipids as a class have many isomers for any given formula and this is reflected in the isomeric composition of the databases at the class and category level. The number of unique entries for a given category is often drastically reduced when redundant isomeric entries are removed for a given formula. This implies that training data sets with large numbers of entries may not necessarily contain more information regarding the mapping of formula to lipid category or class than a smaller more diverse dataset. The addition of the LMISSD addresses this issue partially by introducing more diversity but both the LMSD and LMISSD have large numbers of isomeric entries and the combination of the two also suffers from a similar limitation.

Supplemental Table 3

LMSD + HMDB_non_Lipid Model Performance (Classes)

Category	Precision	Out of Bag Accuracy	Number of Entries	True Positives	True Negatives
Other Fatty Acyls [FA00]	1	0.99895159	19	16	0
Fatty Acids and Conjugates [FA01]	0.85159011	0.96688252	610	482	84
Octadecanoids [FA02]	1	0.99629972	38	7	0
Eicosanoids [FA03]	0.94827586	0.99315449	129	110	6
Docosanoids [FA04]	0.83333333	0.99858156	18	10	2
Fatty alcohols [FA05]	0.98876404	0.99198273	151	88	1
Fatty aldehydes [FA06]	0.88571429	0.99272279	89	31	4
Fatty esters [FA07]	0.9525066	0.97520814	513	361	18
Fatty amides [FA08]	0.95454545	0.99432624	199	189	9
Fatty nitriles [FA09]	1	0.99987666	2	2	0
Fatty ethers [FA10]	NAN	0.99987666	2	0	0
Hydrocarbons [FA11]	1	0.99993833	121	121	0
Oxygenated hydrocarbons [FA12]	0.95238095	0.99346284	86	20	1
Fatty acyl glycosides [FA13]	0.98113208	0.99851989	53	52	1
Other Glycerolipids [GL00]	1	0.9995683	8	8	0
Monoradylglycerols [GL01]	1	0.99907493	18	18	0
Diradylglycerols [GL02]	1	0.99944496	162	162	0
Triradylglycerols [GL03]	1	0.99950663	313	313	0
Glycosylmonoradylglycerols [GL04]	1	0.99987666	2	2	0
Glycosyldiradylglycerols [GL05]	1	0.99950663	29	29	0
Other Glycerophospholipids [GP00]	1	0.99907493	14	12	0
Glycerophosphocholines [GP01]	0.75517241	0.97687327	319	219	71
Glycerophosphoethanolamines [GP02]	0.7	0.97718162	259	161	69
Glycerophosphoserines [GP03]	0.97916667	0.99796485	238	235	5
Glycerophosphoglycerols [GP04]	1	0.99895159	249	246	0
Glycerophosphoglycerophosphates [GP05]	1	0.99987666	2	2	0
Glycerophosphoinositols [GP06]	0.98739496	0.99932162	235	235	3
Glycerophosphoinositol monophosphates [GP07]	1	0.99987666	2	2	0
Glycerophosphoinositol bisphosphates [GP08]	1	0.99993833	1	1	0
Glycerophosphoinositol trisphosphates [GP09]	1	0.99993833	1	1	0
Glycerophosphates [GP10]	0.98765432	0.99845822	241	240	3
Glyceropyrophosphates [GP11]	1	0.99987666	2	2	0
Glycerophosphoglycerophosphoglycerols [GP12]	1	0.99969164	74	74	0
CDP-Glycerols [GP13]	1	0.99993833	18	18	0
Glycosylglycerophospholipids [GP14]	1	0.99950663	5	2	0

Glycerophosphoinositolglycans [GP15]	1	0.99987666	205	205	0
Glycerophosphonocholines [GP16]	NAN	0.99993833	1	0	0
Glycerophosphonoethanolamines [GP17]	NAN	0.99993833	1	0	0
Di-glycerol tetraether phospholipids (caldarchaeols) [GP18]	1	0.99993833	1	1	0
Glycerol-nonitol tetraether phospholipids [GP19]	1	0.99993833	1	1	0
Oxidized glycerophospholipids [GP20]	1	0.99876657	17	9	0
Other Polyketides [PK00]	1	0.99993833	1	1	0
Linear polyketides [PK01]	1	0.9995683	7	7	0
Halogenated acetogenins [PK02]	1	0.99993833	1	1	0
Annonaceae acetogenins [PK03]	1	0.99993833	1	1	0
Macrolides and lactone polyketides [PK04]	1	0.99839655	38	38	0
Ansamycins and related polyketides [PK05]	1	0.99950663	8	8	0
Polyenes [PK06]	1	0.99975331	4	4	0
Linear tetracyclines [PK07]	1	0.99950663	8	8	0
Angucyclines [PK08]	NAN	0.99993833	1	0	0
Polyether antibiotics [PK09]	1	0.99938329	10	10	0
Aflatoxins and related substances [PK10]	1	0.99938329	6	1	0
Cytochalasins [PK11]	1	0.99981499	3	3	0
Flavonoids [PK12]	0.97816961	0.99512797	1168	1165	26
Aromatic polyketides [PK13]	0.95744681	0.99469627	69	45	2
Non-ribosomal peptide/polyketide hybrids [PK14]	1	0.99962997	6	6	0
Phenolic lipids [PK15]	0.97777778	0.99882825	45	44	1
Isoprenoids [PR01]	0.9921671	0.99673142	381	380	3
Quinones and hydroquinones [PR02]	1	0.99808819	35	33	0
Polyprenols [PR03]	1	0.99845822	30	29	0
Hopanoids [PR04]	1	0.99876657	27	26	0
Acylaminosugars [SL01]	1	0.99944496	9	9	0
Acylaminosugar glycans [SL02]	1	0.99981499	3	3	0
Acyltrehaloses [SL03]	1	0.99962997	83	82	0
Other acyl sugars [SL05]	1	0.99950663	8	8	0
Other Sphingolipids [SP00]	1	0.99938329	17	17	0
Sphingoid bases [SP01]	1	0.99864323	67	66	0
Ceramides [SP02]	0.99009901	0.99888992	100	100	1
Phosphosphingolipids [SP03]	1	0.9995683	143	143	0
Phosphonosphingolipids [SP04]	1	0.99987666	2	2	0
Neutral glycosphingolipids [SP05]	0.99094203	0.9862473	553	547	5

Acidic glycosphingolipids [SP06]	0.98664122	0.98686401	520	517	7
Basic glycosphingolipids [SP07]	NAN	0.99987666	1	0	0
Amphoteric glycosphingolipids [SP08]	1	0.99993833	1	1	0
Sterols [ST01]	0.92	0.98606229	348	299	26
Steroids [ST02]	0.91397849	0.99660808	92	85	8
Secosteroids [ST03]	0.89156627	0.98785076	208	148	18
Bile acids and derivatives [ST04]	0.91489362	0.99481961	75	43	4
Steroid conjugates [ST05]	0.98947368	0.99586802	101	94	1
not_lipid	1	1	7587	7587	0

Supplemental Table 4

LMSD + LMISSD + HMDB_non_Lipid Model Performance (Classes)					
Category	Precision	Out of Bag Accuracy	Number of Entries	True Positives	True Negatives
Other Fatty Acyls [FA00]	1	0.99939205	19	16	0
Fatty Acids and Conjugates [FA01]	0.85283688	0.98108214	610	481	83
Octadecanoids [FA02]	1	0.99781855	38	7	0
Eicosanoids [FA03]	0.94827586	0.99617352	129	110	6
Docosanoids [FA04]	0.78571429	0.99921325	18	11	3
Fatty alcohols [FA05]	1	0.99524371	151	87	0
Fatty aldehydes [FA06]	0.93548387	0.9958159	89	29	2
Fatty esters [FA07]	0.94778068	0.98583843	513	363	20
Fatty amides [FA08]	0.94554455	0.99667418	199	191	11
Fatty nitriles [FA09]	1	0.99992848	2	2	0
Fatty ethers [FA10]	NAN	0.99992848	2	0	0
Hydrocarbons [FA11]	1	0.99996424	121	121	0
Oxygenated hydrocarbons [FA12]	1	0.9962808	86	19	0
Fatty acyl glycosides [FA13]	1	0.99903444	53	51	0
Other Glycerolipids [GL00]	1	0.99974967	8	8	0
Monoradylglycerols [GL01]	0.9375	0.99835497	61	45	3
Diradylglycerols [GL02]	0.79207921	0.98458678	492	400	105
Triradylglycerols [GL03]	0.91433022	0.98598148	1242	1174	110
Glycosylmonoradylglycerols [GL04]	1	0.99992848	2	2	0
Glycosyldiradylglycerols [GL05]	1	0.99967815	910	910	0
Other Glycerophospholipids [GP00]	1	0.99942782	14	9	0
Glycerophosphocholines [GP01]	0.71929825	0.96849408	571	287	112
Glycerophosphoethanolamines [GP02]	0.71621622	0.96799342	528	212	84
Glycerophosphoserines [GP03]	0.79683973	0.97300004	517	353	90
Glycerophosphoglycerols [GP04]	0.85281385	0.97975897	525	394	68
Glycerophosphoglycerophosphates [GP05]	1	0.99992848	512	512	0
Glycerophosphoinositols [GP06]	0.83261803	0.9781497	514	388	78
Glycerophosphoinositol monophosphates [GP07]	0.99232246	0.99238279	617	517	4
Glycerophosphoinositol bisphosphates [GP08]	0.91485507	0.9962808	509	505	47
Glycerophosphoinositol trisphosphates [GP09]	0.91485507	0.9962808	509	505	47
Glycerophosphates [GP10]	0.90909091	0.99141723	519	490	49
Glyceropyrophosphates [GP11]	1	0.99992848	2	2	0
Glycerophosphoglycerophosphoglycerols [GP12]	1	0.99974967	315	313	0

CDP-Glycerols [GP13]	1	0.99996424	18	18	0
Glycosylglycerophospholipids [GP14]	1	0.99967815	5	1	0
Glycerophosphoinositolglycans [GP15]	1	0.99971391	205	205	0
Glycerophosphonocholines [GP16]	NAN	0.99996424	1	0	0
Glycerophosphonoethanolamines [GP17]	NAN	0.99996424	1	0	0
Di-glycerol tetraether phospholipids (caldarchaeols) [GP18]	1	0.99996424	1	1	0
Glycerol-nonitol tetraether phospholipids [GP19]	1	0.99996424	1	1	0
Oxidized glycerophospholipids [GP20]	0.88683075	0.91227694	3882	3495	446
Other Polyketides [PK00]	1	0.99996424	1	1	0
Linear polyketides [PK01]	1	0.99974967	7	7	0
Halogenated acetogenins [PK02]	1	0.99996424	1	1	0
Annonaceae acetogenins [PK03]	1	0.99996424	1	1	0
Macrolides and lactone polyketides [PK04]	1	0.9990702	38	38	0
Ansamycins and related polyketides [PK05]	1	0.99971391	8	8	0
Polyenes [PK06]	1	0.99982119	4	4	0
Linear tetracyclines [PK07]	1	0.99971391	8	8	0
Angucyclines [PK08]	NAN	0.99996424	1	0	0
Polyether antibiotics [PK09]	1	0.99964238	10	10	0
Aflatoxins and related substances [PK10]	1	0.99964238	6	1	0
Cytochalasins [PK11]	1	0.99989272	3	3	0
Flavonoids [PK12]	0.97978096	0.99717484	1168	1163	24
Aromatic polyketides [PK13]	1	0.99692451	69	43	0
Non-ribosomal peptide/polyketide hybrids [PK14]	1	0.99974967	6	6	0
Phenolic lipids [PK15]	0.97777778	0.99914172	45	44	1
Isoprenoids [PR01]	0.9921671	0.99806888	381	380	3
Quinones and hydroquinones [PR02]	1	0.99899868	35	33	0
Polyprenols [PR03]	0.96774194	0.99910596	30	30	1
Hopanoids [PR04]	1	0.99928477	27	26	0
Acylaminosugars [SL01]	1	0.99967815	9	9	0
Acylaminosugar glycans [SL02]	1	0.99989272	3	3	0
Acyltrehaloses [SL03]	1	0.99974967	83	82	0
Other acyl sugars [SL05]	1	0.99971391	8	8	0
Other Sphingolipids [SP00]	1	0.99974967	17	17	0
Sphingoid bases [SP01]	1	0.99903444	67	66	0
Ceramides [SP02]	0.99854862	0.99974967	688	688	1
Phosphosphingolipids [SP03]	1	0.99978543	675	675	0

Phosphonosphingolipids [SP04]	1	0.99992848	2	2	0
Neutral glycosphingolipids [SP05]	0.99552372	0.99266888	1118	1112	5
Acidic glycosphingolipids [SP06]	0.98850575	0.9925616	520	516	6
Basic glycosphingolipids [SP07]	NAN	0.99992848	1	0	0
Amphoteric glycosphingolipids [SP08]	1	0.99996424	1	1	0
Sterols [ST01]	0.92	0.99227551	348	299	26
Steroids [ST02]	0.93258427	0.99803312	92	83	6
Secosteroids [ST03]	0.90123457	0.99238279	208	146	16
Bile acids and derivatives [ST04]	0.91489362	0.99696027	75	43	4
Steroid conjugates [ST05]	0.98947368	0.99771126	101	94	1
not_lipid	1	1	7587	7587	0

Tables S3 and S4

Supplemental Table 3 shows the training accuracy and precision for each class-level model in the model collection trained using the LMSD and the HMDB_non_lipid dataset. Excellent precision and accuracy were achieved for most classes; however, some classes have very few examples. For small classes, metrics of accuracy and precision are less useful. During class training, the category of each lipid is known – for the non-lipid category this results in perfect accuracy and precision, because there is only one non-lipid class for the non-lipid category. Each class is trained using a one-against all approach, which inflates the accuracy metric but not precision for each class model. Supplemental Table 4 shows the same results on the LMSD + LMISSD + HMDB_non_lipid dataset. Similar metrics of precision and accuracy were achieved on this larger dataset.

Supplemental Table 5 - LMSD + HMDB_non_lipid Model Performance on Convex Hull (Classes)

LMSD + HMDB_non_lipid Model Performance on Convex Hull (Classes)		
Class	Predictions	% of Hull Formulas
Other Fatty Acyls [FA00]	6	5.41E-06
Fatty Acids and Conjugates [FA01]	848	0.00076495
Octadecanoids [FA02]	6	5.41E-06
Eicosanoids [FA03]	219	0.00019755
Docosanoids [FA04]	4	3.61E-06
Fatty alcohols [FA05]	193	0.0001741
Fatty aldehydes [FA06]	20	1.80E-05
Fatty esters [FA07]	466549	0.42085463
Fatty amides [FA08]	4715	0.00425321
Fatty nitriles [FA09]	2	1.80E-06
Hydrocarbons [FA11]	254	0.00022912
Oxygenated hydrocarbons [FA12]	22	1.98E-05
Fatty acyl glycosides [FA13]	839	0.00075683
Other Glycerolipids [GL00]	13	1.17E-05
Monoradylglycerols [GL01]	8	7.22E-06
Diradylglycerols [GL02]	1268	0.00114381
Triradylglycerols [GL03]	5206	0.00469612
Glycosylmonoradylglycerols [GL04]	2	1.80E-06
Glycosyldiradylglycerols [GL05]	397	0.00035812
Other Glycerophospholipids [GP00]	153	0.00013801
Glycerophosphocholines [GP01]	7248	0.00653812
Glycerophosphoethanolamines [GP02]	3514	0.00316983
Glycerophosphoserines [GP03]	7440	0.00671132
Glycerophosphoglycerols [GP04]	4335	0.00391042
Glycerophosphoglycerophosphates [GP05]	1	9.02E-07
Glycerophosphoinositols [GP06]	7032	0.00634328
Glycerophosphoinositol monophosphates [GP07]	2	1.80E-06
Glycerophosphoinositol bisphosphates [GP08]	1	9.02E-07
Glycerophosphoinositol trisphosphates [GP09]	1	9.02E-07
Glycerophosphates [GP10]	3971	0.00358208

Glyceropyrophosphates [GP11]	2	1.80E-06
Glycerophosphoglycerophosphoglycerols [GP12]	15312	0.01381232
CDP-Glycerols [GP13]	9047	0.00816093
Glycosylglycerophospholipids [GP14]	1	9.02E-07
Glycerophosphoinositolglycans [GP15]	486743	0.4390708
Oxidized glycerophospholipids [GP20]	14	1.26E-05
Other Polyketides [PK00]	1	9.02E-07
Linear polyketides [PK01]	1	9.02E-07
Macrolides and lactone polyketides [PK04]	260	0.00023454
Ansamycins and related polyketides [PK05]	21	1.89E-05
Polyenes [PK06]	1	9.02E-07
Linear tetracyclines [PK07]	4	3.61E-06
Polyether antibiotics [PK09]	2	1.80E-06
Cytochalasins [PK11]	1	9.02E-07
Flavonoids [PK12]	81057	0.07311818
Aromatic polyketides [PK13]	76	6.86E-05
Non-ribosomal peptide/polyketide hybrids [PK14]	2029	0.00183028
Phenolic lipids [PK15]	7	6.31E-06
Isoprenoids [PR01]	6838	0.00616828
Quinones and hydroquinones [PR02]	2234	0.0020152
Polyprenols [PR03]	2207	0.00199084
Hopanoids [PR04]	168	0.00015155
Acylaminosugars [SL01]	48	4.33E-05
Acylaminosugar glycans [SL02]	1151	0.00103827
Acyltrehaloses [SL03]	540	0.00048711
Other acyl sugars [SL05]	6	5.41E-06
Other Sphingolipids [SP00]	339	0.0003058
Sphingoid bases [SP01]	277	0.00024987
Ceramides [SP02]	2601	0.00234625
Phosphosphingolipids [SP03]	12702	0.01145795
Phosphonosphingolipids [SP04]	4	3.61E-06
Neutral glycosphingolipids [SP05]	1766761	1.5937223
Acidic glycosphingolipids [SP06]	3190039	2.87760274
Amphoteric glycosphingolipids [SP08]	1	9.02E-07
Sterols [ST01]	9421	0.0084983
Steroids [ST02]	30	2.71E-05
Secosteroids [ST03]	1508	0.0013603

Bile acids and derivatives [ST04]	60	5.41E-05
Steroid conjugates [ST05]	5728	0.00516699
no_class	31556423	28.4657489
not_lipid	74621680	67.3131427

Supplemental Table 6 - LMSD + LMISSD + HMDB_non_lipid Model Performance on Convex Hull (Classes)

LMSD + LMISSD + HMDB_non_lipid Model Performance on Convex Hull (Classes)		
Class	Predictions	Percentage
Other Fatty Acyls [FA00]	5	4.51E-06
Fatty Acids and Conjugates [FA01]	524	0.00047268
Octadecanoids [FA02]	7	6.31E-06
Eicosanoids [FA03]	217	0.00019575
Docosanoids [FA04]	4	3.61E-06
Fatty alcohols [FA05]	122	0.00011005
Fatty aldehydes [FA06]	18	1.62E-05
Fatty esters [FA07]	386306	0.34847072
Fatty amides [FA08]	3939	0.00355321
Fatty nitriles [FA09]	2	1.80E-06
Hydrocarbons [FA11]	259	0.00023363
Oxygenated hydrocarbons [FA12]	24	2.16E-05
Fatty acyl glycosides [FA13]	537	0.00048441
Other Glycerolipids [GL00]	11	9.92E-06
Monoradylglycerols [GL01]	34	3.07E-05
Diradylglycerols [GL02]	2726	0.00245901
Triradylglycerols [GL03]	11297	0.01019056
Glycosylmonoradylglycerols [GL04]	2	1.80E-06
Glycosyldiradylglycerols [GL05]	28846	0.02602079
Other Glycerophospholipids [GP00]	165	0.00014884
Glycerophosphocholines [GP01]	7739	0.00698103
Glycerophosphoethanolamines [GP02]	2315	0.00208827
Glycerophosphoserines [GP03]	6301	0.00568387
Glycerophosphoglycerols [GP04]	4755	0.00428929
Glycerophosphoglycerophosphates [GP05]	17058	0.01538732
Glycerophosphoinositols [GP06]	5926	0.0053456
Glycerophosphoinositol monophosphates [GP07]	20099	0.01813048
Glycerophosphoinositol bisphosphates [GP08]	19207	0.01732584
Glycerophosphoinositol trisphosphates [GP09]	189305	0.17076424

Glycerophosphates [GP10]	9926	0.00895384
Glyceropyrophosphates [GP11]	2	1.80E-06
Glycerophosphoglycerophosphoglycerols [GP12]	10456	0.00943193
CDP-Glycerols [GP13]	4178	0.0037688
Glycerophosphoinositolglycans [GP15]	551629	0.49760179
Oxidized glycerophospholipids [GP20]	97265	0.08773875
Other Polyketides [PK00]	1	9.02E-07
Linear polyketides [PK01]	1	9.02E-07
Macrolides and lactone polyketides [PK04]	417	0.00037616
Ansamycins and related polyketides [PK05]	20	1.80E-05
Polyenes [PK06]	1	9.02E-07
Linear tetracyclines [PK07]	4	3.61E-06
Polyether antibiotics [PK09]	2	1.80E-06
Cytochalasins [PK11]	1	9.02E-07
Flavonoids [PK12]	110362	0.09955301
Aromatic polyketides [PK13]	87	7.85E-05
Non-ribosomal peptide/polyketide hybrids [PK14]	2578	0.00232551
Phenolic lipids [PK15]	6	5.41E-06
Isoprenoids [PR01]	4532	0.00408813
Quinones and hydroquinones [PR02]	2587	0.00233363
Polyprenols [PR03]	1806	0.00162912
Hopanoids [PR04]	146	0.0001317
Acylaminosugars [SL01]	23	2.07E-05
Acylaminosugar glycans [SL02]	598	0.00053943
Acyltrehaloses [SL03]	143	0.00012899
Other acyl sugars [SL05]	6	5.41E-06
Other Sphingolipids [SP00]	316	0.00028505
Sphingoid bases [SP01]	381	0.00034368
Ceramides [SP02]	11981	0.01080757
Phosphosphingolipids [SP03]	36322	0.03276458
Phosphonosphingolipids [SP04]	3	2.71E-06
Neutral glycosphingolipids [SP05]	1765763	1.59282204
Acidic glycosphingolipids [SP06]	5649731	5.09638954
Amphoteric glycosphingolipids [SP08]	1	9.02E-07
Sterols [ST01]	7910	0.00713529
Steroids [ST02]	33	2.98E-05
Secosteroids [ST03]	1253	0.00113028

Bile acids and derivatives [ST04]	50	4.51E-05
Steroid conjugates [ST05]	5385	0.00485759
no_class	31653829	28.5536148
not_lipid	73562707	66.3578868

Tables S5 and S6

Lipid classes, in addition to categories, were predicted for every formula within the hull. For many classes, more formulas were predicted for each category than existed in the training dataset, indicating that the models have generalized beyond the training dataset. However, some classes, which had a limited number of examples in the training dataset, only had a handful of predictions (e.g. amphoteric glycosphingolipids have one prediction but only one example in the training data). As seen previously, the selection of training data does vary the number of predictions per class, but in general, the trends are the same between the LMSD trained models and the LMISSD trained models. As seen at the category-level, sphingolipids are frequently predicted, likely overpredicted, at the class level. Fortunately, this over prediction occurs for only a subset of sphingolipids, namely acidic and neutral glycosphingolipids, suggesting that predictions to the classes are less trustworthy than predictions to other classes of sphingolipids.

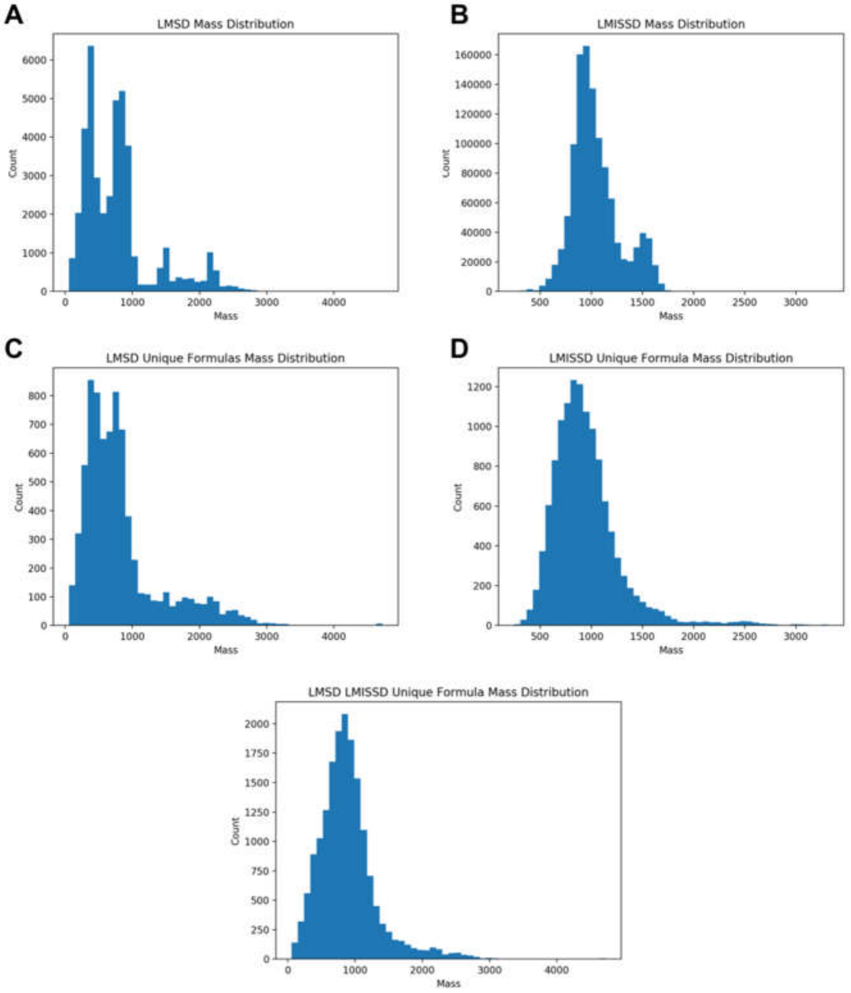

Supplemental Figure 1: Training Set Mass Distributions

Both the LMSD and LMISSD are heavily biased towards lipids with a mass below 1200 m/z (Panels A and B respectively). This effect becomes clearer once entries are deduplicated to yield only unique formulas (Panels C and D). Some entries exist out to 3000+ m/z but the bulk of the formulas still reside in the sub 1200 mass range. When combined, the bias is still present in the combined set of unique formulas (Panel E). Additionally, the LMISSD does not have entries from all the lipid categories.

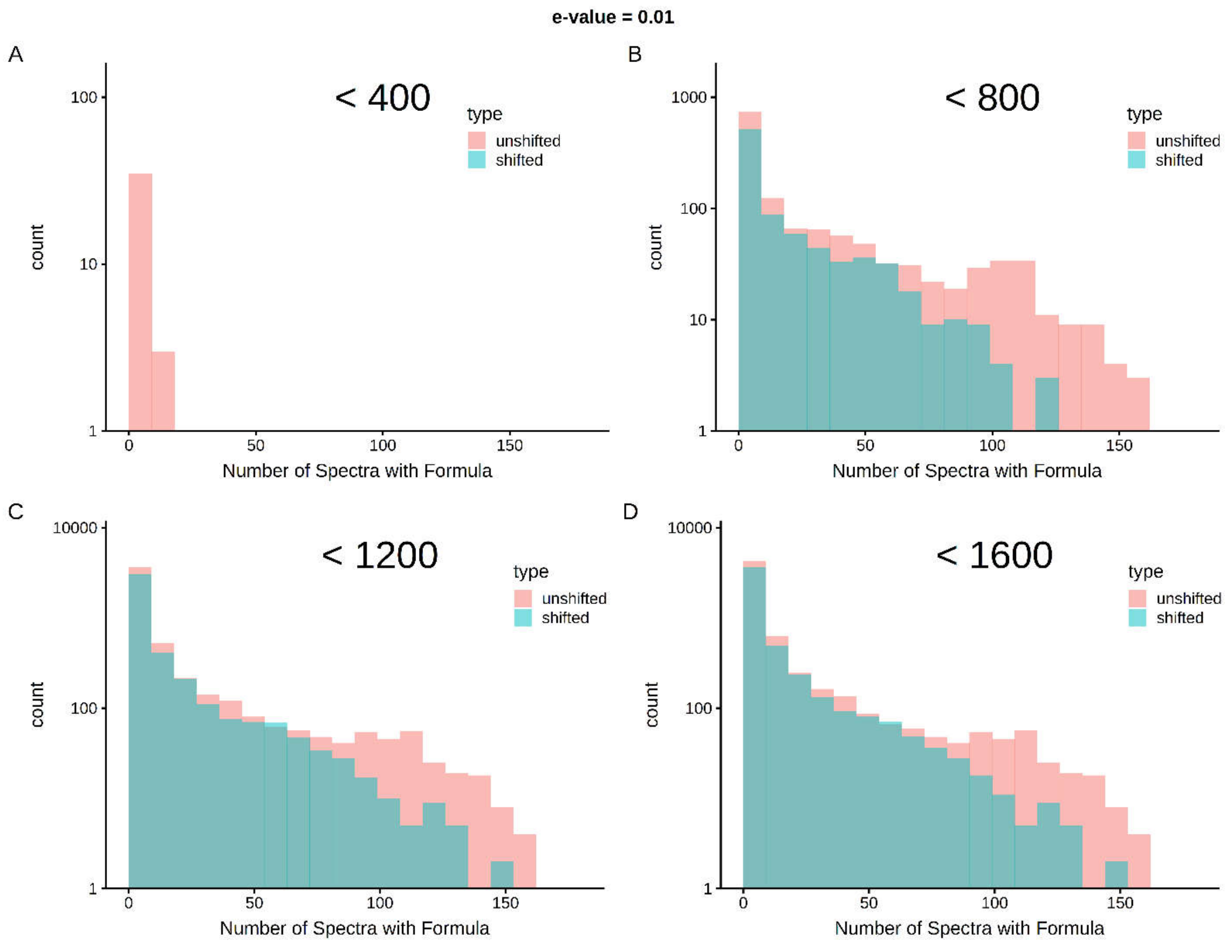

Supplemental Figure 2: Cross-Sample Correspondence with Lipid Classification at E-value <= 0.01

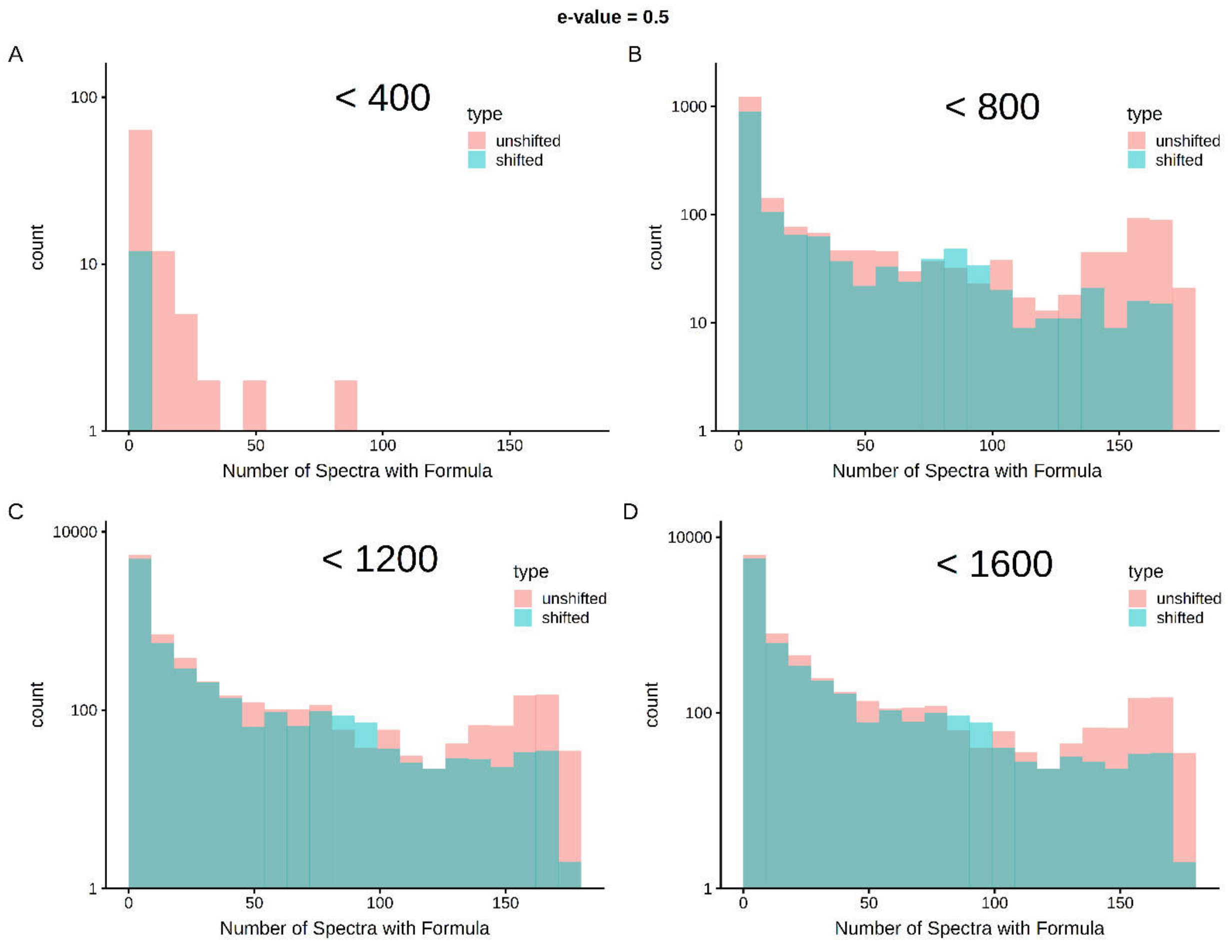

Supplemental Figure 3: Cross-Sample Correspondence with Lipid Classification at E-value <= 0.5

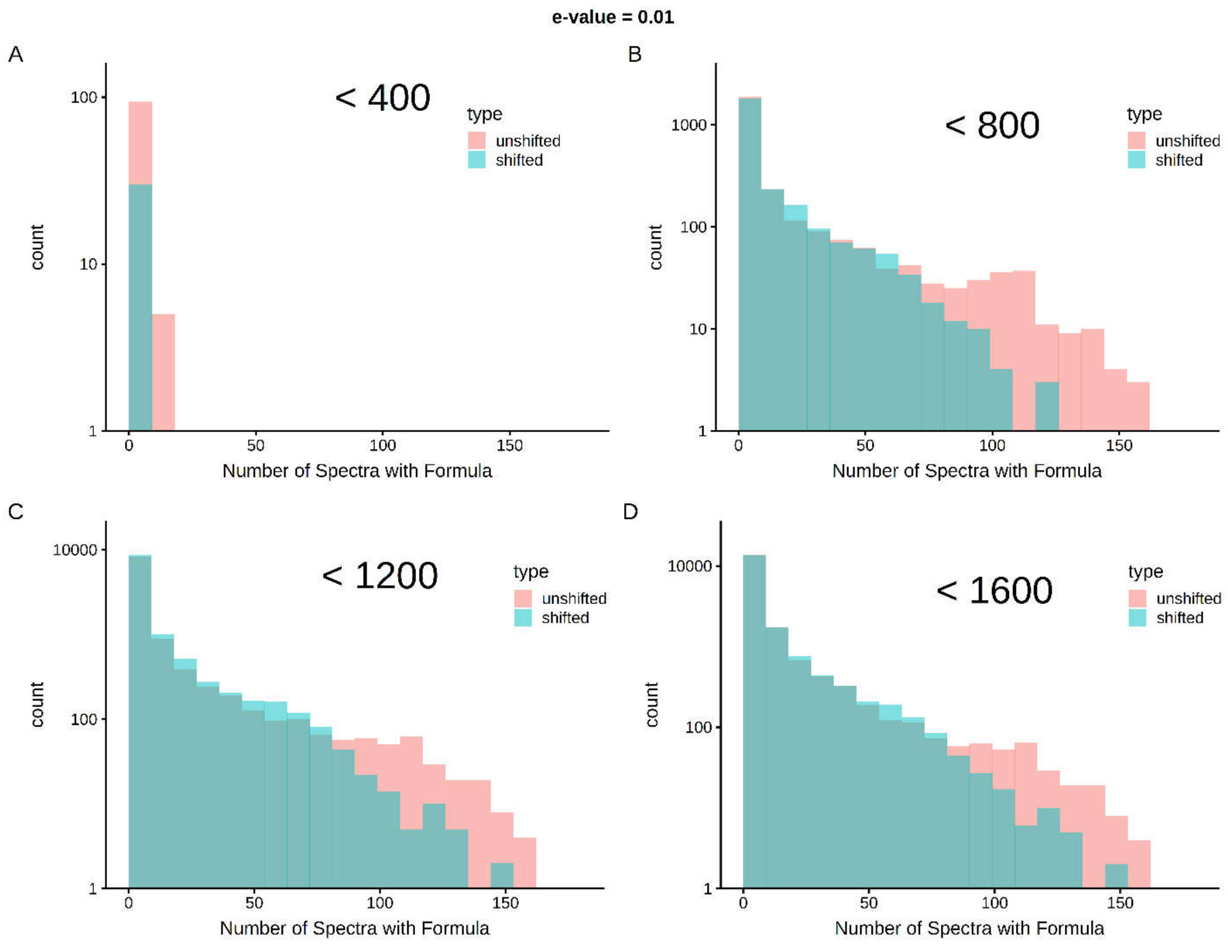

Supplemental Figure 4: Cross-Sample Correspondence at E-value <= 0.01

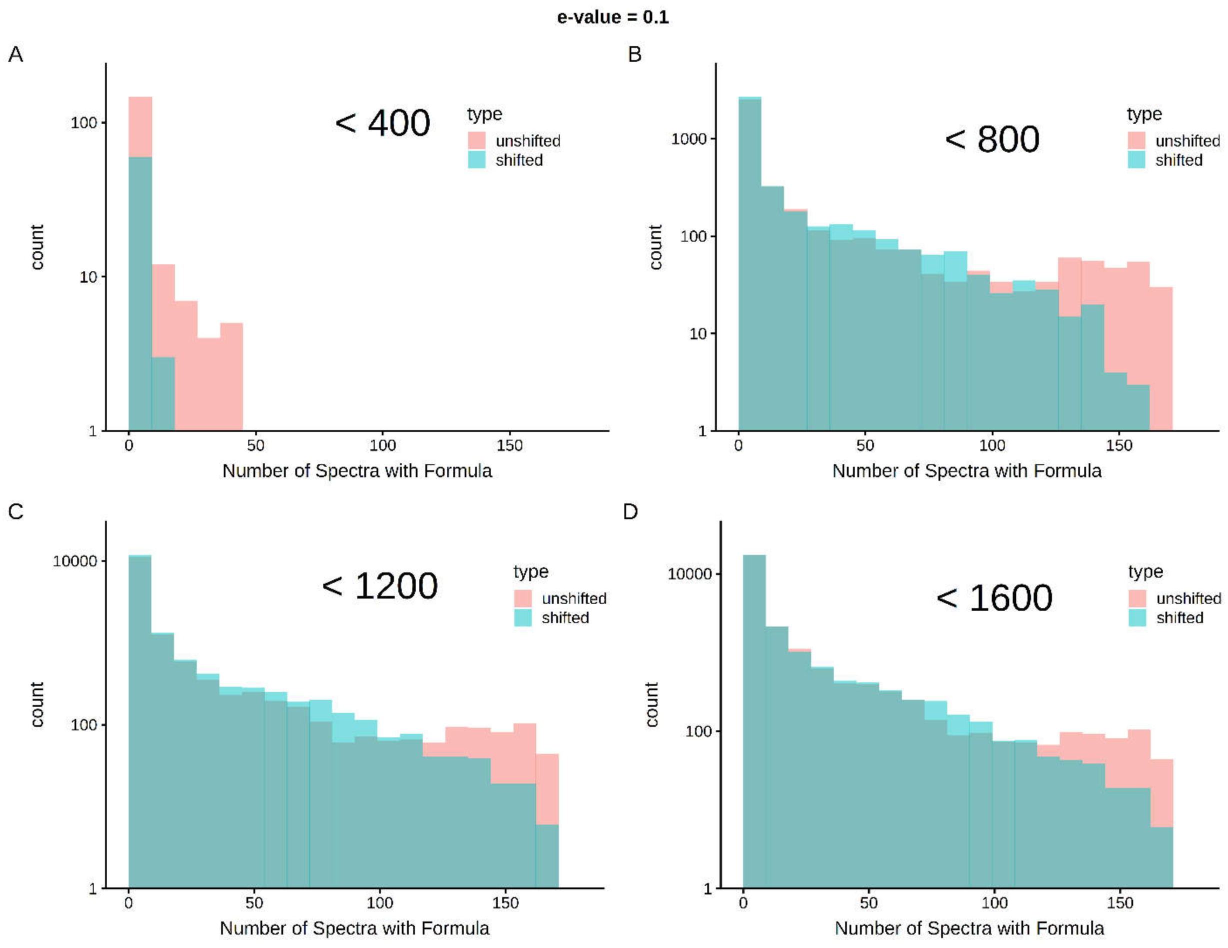

Supplemental Figure 5: Cross-Sample Correspondence at E-value <= 0.1

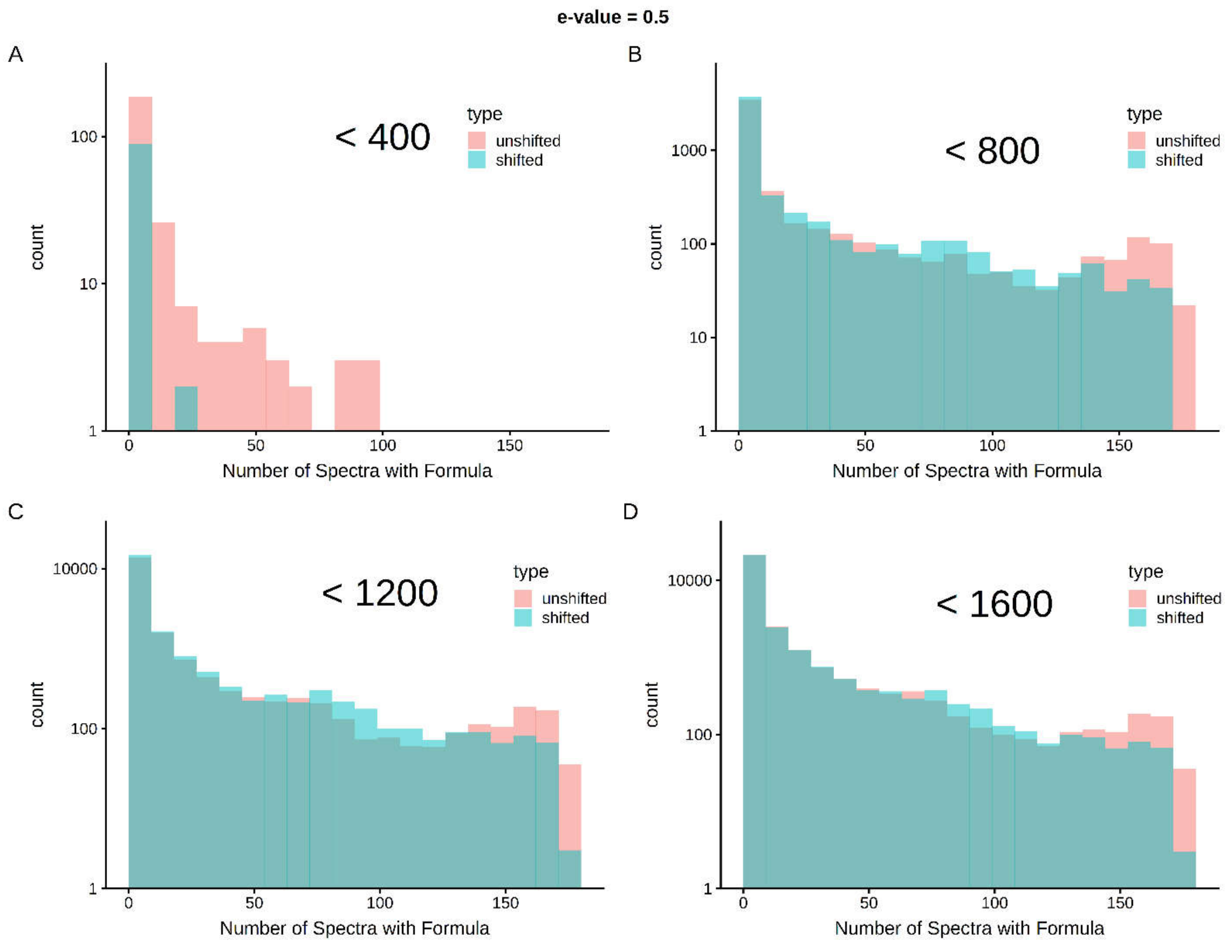

Supplemental Figure 6: Cross-Sample Correspondence at E-value <= 0.5
